# Focus Tracking System for Femtosecond Laser Machining using Low Coherence Interferometry

**DOI:** 10.1038/s41598-019-40749-6

**Published:** 2019-03-12

**Authors:** Marcus Paulo Raele, Lucas Ramos De Pretto, Wagner de Rossi, Nilson Dias Vieira, Ricardo Elgul Samad

**Affiliations:** Nuclear and Energy Research Institute, IPEN-CNEN/SP, Av. Prof. Lineu Prestes, 2242, Cidade Universitária, 05508-000, São Paulo, SP Brazil

## Abstract

We designed a real time, single-laser focus tracking system using low coherence properties of the machining femtosecond laser itself in order to monitor and correct the sample position relative to the focal plane. Using a Michelson Interferometer, the system collects data arising from part of the beam backscattered at the ablation spot. The data is analyzed by a custom software for position correction (employing an XYZ automated translation stage). With the focus tracking enabled we were able to etch channels with a stable cross-section profile on a bovine tooth with relief amplitude tens of times greater than the Rayleigh length of the system, keeping the sample inside the confocal parameter during most of the processing time. Moreover, the system is also capable of monitoring crater depth evolution during the ablation process, allowing for material removal assessment.

## Introduction

Femtosecond laser machining (FLM) has received a lot of interest^1^ due to high precision etching with minimized thermal effects^2^. FLM needs tight and constant focusing of the beam on the surface under treatment to maintain the laser intensity and a precise lateral resolution. In most laser machining systems, this restricts processing to samples with flat profiles, and a wide range of subjects cannot be rectified or polished without losing their primary functions. To extend their applicability, machining systems should compensate for non-flat and irregular samples. We present here results of a focus tracking system for femtosecond pulses based on low coherence interferometry (LCI), using the machining beam itself as a probe. This approach enabled the etching of grooves on a bovine tooth with surface curvature amplitude greater than the confocal parameter (CP) of the system, maintaining the groove characteristics stable. Our setup also allowed monitoring the ablation crater evolution in real time.

In order to cope with variations when machining with lasers, the system should compensate for deviations in the lens-to-sample distance, keeping the sample surface on the system focus and its spot size constant. Several approaches have been proposed to actively perform this task^3,4^. Cao *et al*. summarized many of the leading methods on their work^4^.

**Figure 1:**
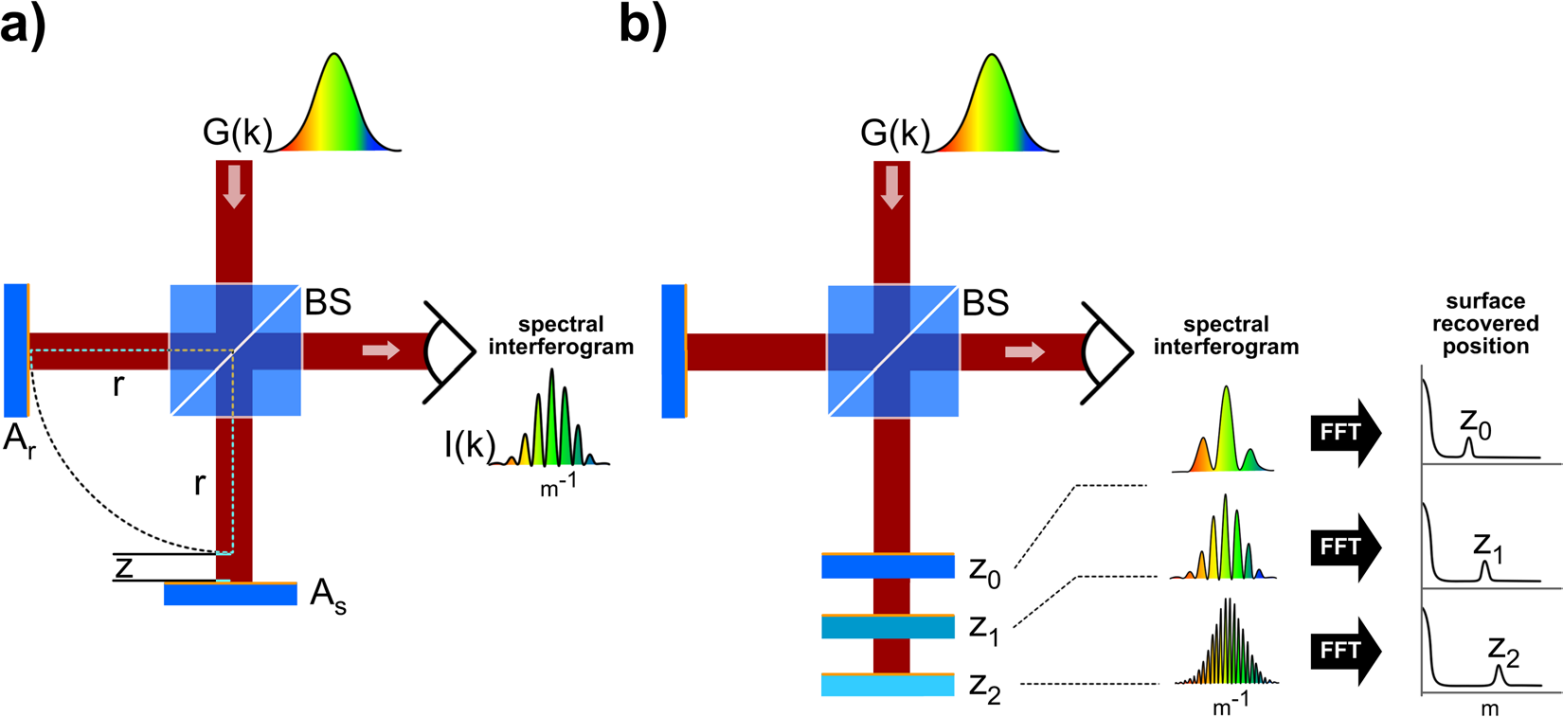
(**a**) LCI setup: a light source, with spectral distribution *G*(*k*), is divided by a beam splitter (BS) and part of the beam is directed to a reference arm with reflectivity *A*_*r*_, while the other part goes to the sample arm with reflectivity *A*_*s*_. The arms are set up with an OPD of *z*. The reflected beams are recombined, producing the spectral distribution *I*(*k*), which is analyzed by a spectrometer. (**b**) Since the intensity is modulated by the OPD, the system can recover the surface position (relative to the Reference known position) through a FFT of the interferometric signal.

Probing the lens-to-sample distance may be performed optically using interferometric methods such as Low Coherence Interferometry^5^. In this technique, a beam from a broadband light source is split, by a Beam Splitter (BS), into two different optical paths (usually mounted on a Michelson Interferometer as illustrated on Fig. 1a) that are later recombined and detected, producing an interferometric signal. One of the optical paths is defined as the “Reference Arm” and contains a mirror with reflectivity *A*_*r*_ positioned at a distance *r* from the BS; similarly, the other arm (“Sample Arm”) has a mirror of reflectivity *A*_*s*_ at a distance *r* + *z*. In this case, *z* represents the optical path difference, OPD. As the light makes a round trip to each mirror, there’s a factor 2 increasing the OPD. For simplicity, we assume a 50:50 beam splitter; refractive index of 1 for the whole system; mirrors with perfect reflectivity; and a light source with a spectral distribution *G*(*k*). The intensity at the detector can then be described as a function of the wavenumber *k*, and OPD *z*:

1 $$I(k,z)={|\frac{G(k)}{2}{A}_{r}{e}^{(i2knr)}+\frac{G(k)}{2}{A}_{s}{e}^{(i2kn(r+z))}|}^{2}$$

2 $$I(k,z)={(\frac{G(k)}{2})}^{2}\cdot (2+2{\int }_{z=0}^{\infty }\cos (2kz)dz)$$

The influence of the OPD^6,7^ on the signal is easily seen when analyzing the real part of Equation 1 and applying the Euler relationship $({e}^{\pm i\theta }=\,\cos \,\theta \pm i\,\sin \,\theta )$. The OPD is encoded in a cosine frequency univocally for *z* > 0, as shown in Equation 2. Hence, the intensity on the detector will have a spectrum with an envelope given by G(*k*) and a periodic pattern defined by *z* (Fig. 1a). Computationally, the cosine frequency - and, thus, the OPD - can be recovered by a Fast Fourier Transform (FFT) of the signal. The obtained OPD, therefore, provides direct information of the Sample position relative to the Reference (Fig. 1b). Also noteworthy, a broader G(*k*), will result in a narrower FFT[G(k)], which is associated with the system’s axial resolution^6,8^.

A LCI measurement with the machining beam is feasible due to the broadband spectrum of femtosecond pulses^9^. Since a fraction of the incident beam gets invariably reflected from the sample surface when machining, it may act as the mirror in the Sample Arm. Then, one can monitor the position of the surface under machining by the variations in the periodic structure of the resulting spectral signal, in the same way shown in Fig. 1b.

The axial resolution for LCI setups depends on the coherence length^9^ of the source. For a typical femtosecond (Ti:Sapphire) amplified system with a 40 nm spectral bandwidth (FWHM), the resulting axial resolution is about 6 μm. This value is smaller than the confocal parameter for a 10 mm Gaussian beam focused by a 25 mm effective focal length (EFL) lens, which is around 12 μm, denoting that such a resolution is enough to keep the sample inside the confocal range of the system.

The objective of the present work is to demonstrate the viability of a laser machining system assisted by a LCI setup to track the surface relief and correct the sample position in relation to the focal plane in real time

## Methods

### System setup

**Figure 2:**
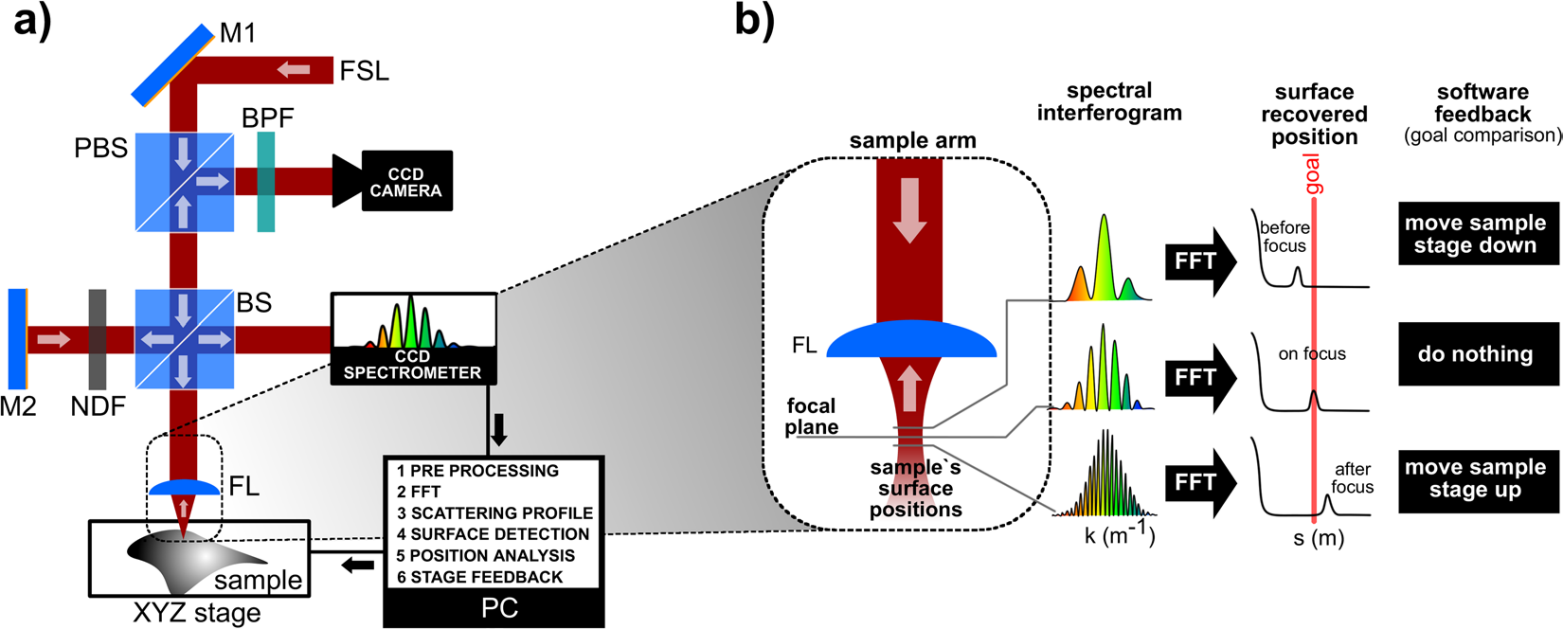
(**a**) The experimental setup: femtosecond laser (FSL) pulses are transmitted through the polarized beam splitter (PBS) and then divided by a 50:50 beam splitter (BS). Part of the FSL radiation is directed to a reference arm after passing a neutral density filter (NDF), and is reflected by the mirror (M2) towards the spectrometer. The other portion of the FSL is focused on the sample’s surface, performing the ablation, and a fraction is backscattered to the BS and spectrometer. The back reflected light also reaches the PBS and, after going through a bandpass filter (BPF), is imaged through a CCD camera for visual inspection. The sample is positioned on a XYZ translation stage. (**b**) The processing diagram for the system, where a “goal” OPD position - which coincides with the focus of the system - is defined. During machining, the software continuously analyzes the OPD deviation from the “goal”, and decides whether an action on the XYZ translation stage should be carried out.

To make use of LCI for focus correction as proposed, we assembled an optical setup according to Fig. 2a. The femtosecond laser (FSL) used is an amplified Ti:Sapphire system (Femtopower Compact Pro HR/HP, from Femtolasers), generating 25 fs pulses at 4 kHz repetition rate, centered at 785 nm with 40 nm of spectral bandwidth (FWHM), resulting in a axial resolution (coherence length) of 6.8 µm. A polarized beam splitter (PBS) allowed the polarized pulses to travel with minimal losses to a 50:50 non-polarized beam splitter (BS) used in the Michelson Interferometer. As the surface under machining (Sample Arm) would not reflect the FSL efficiently like a metallic Reference Mirror M2, a variable neutral density filter (NDF) was used to balance the light intensity in both arms. M2 was mounted on a translation stage so the *r* value (Fig. 1a) could be adjusted. At the sample arm, a 25 mm EFL lens was used with a numeric aperture of 2.5, resulting in a 4.7 μm beam waist w_0_ (for M^2^ = 1.6) and confocal parameter of 49.6 μm. The reflected light is directed towards the spectrometer through the BS and some fraction goes to a CCD camera (for visual inspection purposes) through the PBS.

### Determining the focus and defining goal position

To find out the focal plane for the system a series of grooves were etched on a glass slide while varying its distance to the lens. In this step, no interferometry measurements were made. Optical and scanning electron microscopy were applied to evaluate the visual aspect and machining quality of the grooves, allowing to determine the focal plane.

The glass slide was positioned at the obtained focal plane, the laser intensity was decreased and the LCI was switched on. The slide was kept still and the M2 translation stage was used to adjust *r* so that the focal position coincided with an OPD of 300 μm (goal). This method is fully described in a previous work^10^.

### Focus tracking system and axial correction

Based on real-time analysis of the LCI feedback (Fig. 2b), an algorithm was developed in Labview™ for acting on the XYZ translation stage in order to keep the sample surface at the defined goal position. The algorithm continuously evaluates the current OPD (surface position) and accounts for the amount of deviation from the goal as well as the trend (below or above focal plane) to determine the necessary correction on the axial (Z) axis.

**Figure 3:**
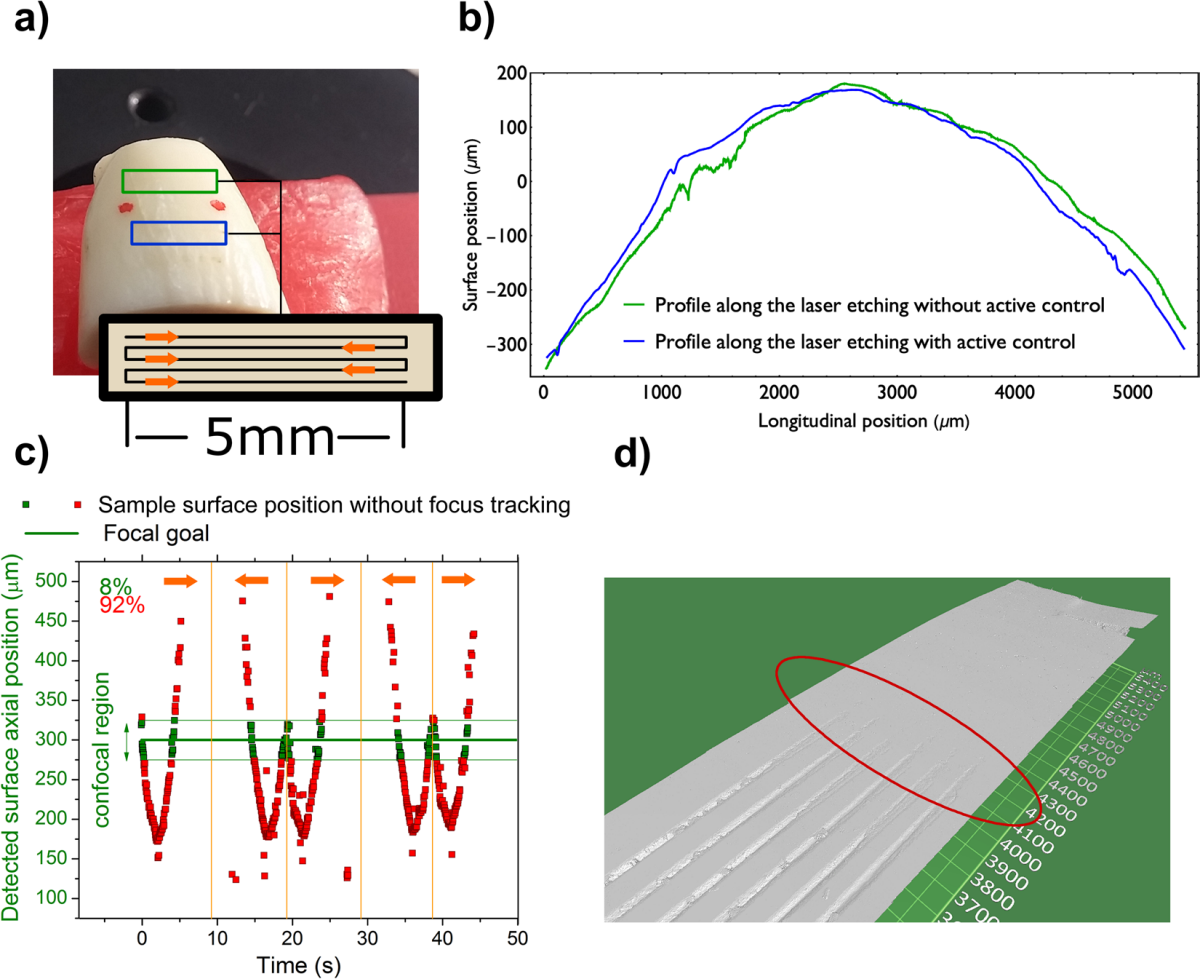
(**a**) Bovine tooth sample. The machined regions with and without active focus tracking are highlighted (blue and green boxes, respectively), the raster pattern is illustrated in the black box and the arrows indicate the laser path. (**b**) Tooth surface profiles showing the curvature along the raster path with (blue) and without (green) focus tracking. There is a total axial displacement of ∼1000 μm for both regions. (**c**) Surface position (during raster) over time. Square dots represent the detected axial position of the sample surface (relative to the OPD). The surface curvature (axial displacements in the graph) repeats itself over time as the raster goes back and forth. The focal plane (thick solid green line at 300 μm) and its confocal parameter (lighter green lines) are shown for reference. For ease of interpretation, the dots inside confocal parameter are colored green, while those outside (faulty) are red. The vertical orange lines indicate when the raster motion changed direction, which is represented by the orange arrows. (**d**) Optical profilometry image showing the ceasing of the ablation process after 4000 μm of raster length, shorter than desired.

Aiming to demonstrate the potential use in clinical/medical applications^11^, the focus tracking system was tested on a bovine tooth. The sample was placed at the focal plane of the system and two separate regions were studied (Fig. 3a): with (blue box) and without (green box) active focus tracking control. The sample machining was performed in a raster pattern (0.5 mm/s) of 5 lines with 5 mm length, spaced by 50 μm, laser pulse energy of (9.05 ± 0.18) µJ and fluence equal to 13.5 J/cm^2^. The machined regions were later analyzed by optical profilometry (Zygo ZeGage).

### Crater ablation evolution monitoring

With the LCI data it is also possible to monitor in real-time the evolution of the crater being ablated on the surface, since the material removal will affect the OPD. In order to do so, the sample is kept static (no raster movement is performed), and the focus tracking system is disabled. The sample LCI data is recorded through time at the spot being etched.

As a demonstration of the ablation monitoring capability, a layered metal sample (Brazilian coin – steel coated with bronze) was studied. It was placed at the focal plane and processed with (11.72 ± 0.22) µJ femtosecond pulses, corresponding to a fluence of 16.5 J/cm^2^. A single spot on the sample was studied throughout 2500 overlapped laser pulses, with its LCI profile recorded.

## Results and Discussion

### Focus tracking system

Tooth surface profile measurements (Fig. 3b) at the highlighted regions are similar and indicate a total axial displacement on the order of 1000 μm (from −300 μm, peaking at +200 μm and going back down to −300 μm) along the raster path, much larger than the system confocal parameter.

On the first run, with focus tracking disabled, the system recorded the axial surface position, shown in Fig. 3c, and revealed that the surface was inside the confocal parameter only 8% of the time, leading to the expected faulty machining, as one can see in Fig. 3d.

**Figure 4:**
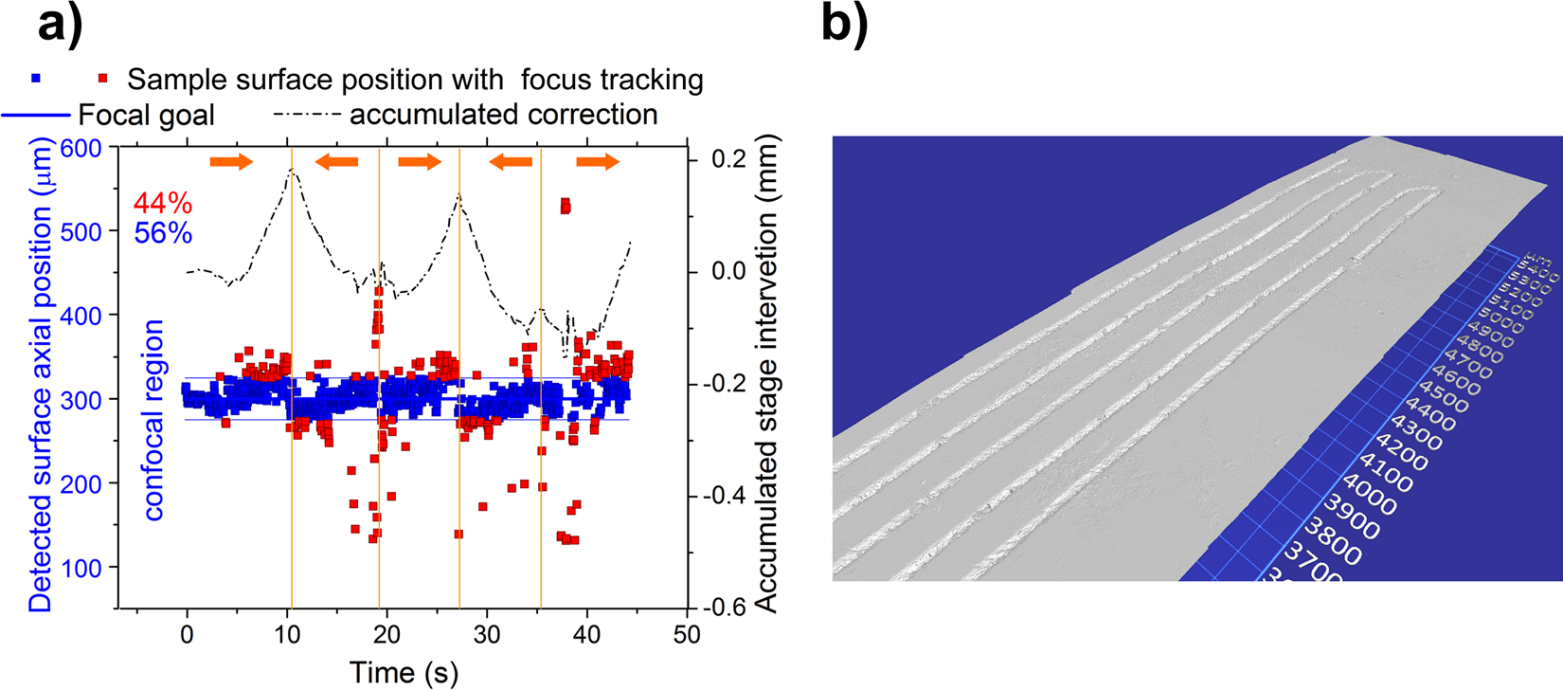
(**a**) Surface position over time during the machining with active focus tracking. The position of the axial translation stage, acting to correct the sample position is shown by the dash&dot black line (right Y axis). Square dots represent the detected position of the sample surface; the focal plane and its confocal parameter (solid blue lines, thicker and thinner, respectively) are shown for reference. The dots inside confocal parameter are colored blue, and outside are red. The vertical orange lines indicate when the raster motion changed direction, which is indicated by the orange arrows. (**b**) Optical profilometry image, showing that machining occurred along the whole raster path.

With active focus tracking enabled the system intervened, moving the axial translation stage (Fig. 4a, dash&dot line), keeping the sample inside the confocal parameter most of the time, enabling machining to occur efficiently over the whole raster pattern (Fig. 4b).

The focus tracking results presented some data points that are far from the desired region (confocal parameter). These instances are related to the raster direction change, in which the lateral motion reverses direction (creating the zigzag pattern in Fig. 3a). When that occurs, any feature from the sample that happens to be causing positive shifts on the OPD during the end of a given raster line will abruptly lead to negative shifts when the subsequent one starts (and vice versa). Owing to that, in the occasions of raster motion reversal, indicated by the vertical orange lines in Fig. 4a, the system lost track of the surface, overshot the correction and needed a few iterations to compensate and regain proper control. Furthermore, due to hardware limitations on the spectrometer, each interferogram collected was an integration of four consecutive laser shots. Different laser shots may come from varying OPD’s, resulting in a blurred interferogram, leading the system astray.

Nonetheless, the system maintained the sample surface within the confocal parameter on 56% of the events, with overall smaller excursions outside the confocal range when compared with the machining without active control. This is indicated in Table 1, which contains the percentages of events where the position of the sample surface (S) fell within different multiples of the confocal parameter. In some events the surface was not identified by the system, due to a number of different causes, such as interferometric signal blurring (generated by the long integration time), weak signal or excursions beyond the system detection range. Those points are labeled “unclassified”, and Table 1 also presents results excluding such points, in which case the focus tracking system was able to maintain the surface inside the confocal range during 79% of the events. One may also note that excursions outside the confocal range are smaller when the tracking system is enabled, with 68% of the events (94% without “unclassified” points) within 2 CP against 14% (32% without “unclassified”) with the system disabled. Considering the algorithm logic, the “unclassified” points will tend to remain close to the CP region with the correction system enabled, and will be caused mainly by blurring or noise. When the system is disabled, the surface moves away from the optical optimal range, implying in weak signal or even exceeding the system detection range, suggesting that most of the “unclassified” points fall out of the CP region.

**Table 1:** Comparison of the performance (percentages of events recorded) with and without the focus tracking system.

Focus Tracking	All data values				Excluding “Unclassified”		
	S < 1 CP	1 CP < S < 2 CP	2 CP < S	Unclassified	S < 1 CP	1 CP < S < 2 CP	2 CP < S
Enabled	56%	12%	4%	28%	79%	16%	6%
Disabled	8%	6%	29%	56%	18%	15%	68%

Data was divided into groups according to surface position (S) being recorded within one confocal parameter (S < 1 CP); between one and two CP (1 CP < S < 2 CP); and outside 2 CP (2 CP < S). Unclassified events happened when the system could not properly locate the surface.

**Figure 5:**
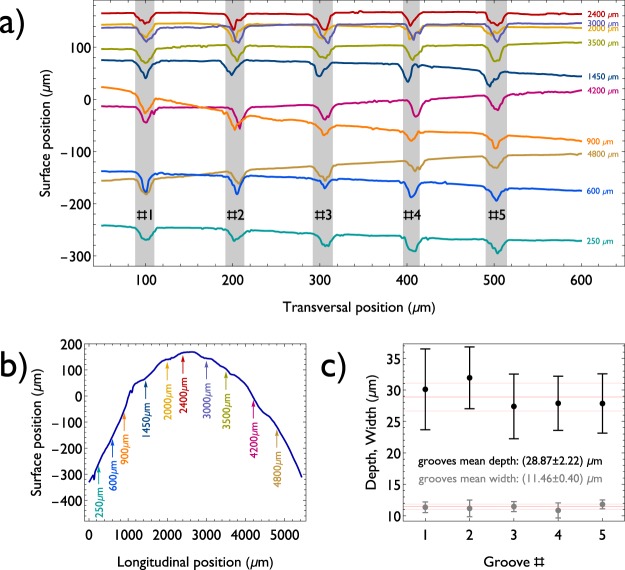
(**a**) Transversal profiles of the machined sample, plotted with their absolute position (height), showing the machined grooves’ shapes at the different longitudinal locations, indicated by arrows in (**b**); the numbers on the gray regions identify each groove. (**b**) Longitudinal profile of the tooth (parallel to the grooves) with arrows indicating the positions of the profiles show in (**a**), highlighting a height variation of 500 μm; the curvature of the surface, seen here, explains the positioning of the profiles in (**a**). (**c**) Average width (gray) and depth (black) of the five grooves obtained from the 10 measured longitudinal positions, demonstrating a stable machining across grooves.

The performance of our system enabled a steady and uninterrupted ablation process, as seen in Fig. 4b and further evidenced by analyzing the machined grooves. From a topographic map obtained by optical profilometry, 10 transversal profiles were acquired, intercepting all five grooves etched with active focus tracking. The profiles are displayed in Fig. 5a, according to their surface position, while Fig. 5b indicates their location on the sample surface (the same surface profile previously shown in Fig. 3b). A 3D map indicating all the grooves and profiles is provided in the supplementary information material (Fig. S1). The gray regions in Fig. 5a identify each one of the five grooves, and their shapes can be clearly seen in the profiles, all exhibiting similar depths and widths. To quantify these similarities, the mean values for width (gray) and depth (black) of each groove were calculated (from the 10 profiles) and are plotted in Fig. 5c. The width of a given groove in a profile was estimated by calculating its second moment (variance), defined by^12^:

3 $${\mu }_{2}=\frac{\int {(x-{\mu }_{1})}^{2}h(x)dx}{\int h(x)dx},$$

where *x* is the spatial coordinate, *h*(*x*) is the surface position (height) and *μ*_1_ is the groove first moment (center of gravity):

4 $${\mu }_{1}=\frac{\int {\rm{x}}\,h(x)dx}{\int h(x)dx}.$$

These moments were used since the groove’s shapes vary and cannot be well described by analytical functions. Summations were used instead of integrals due to the discrete sampling. Further details about the grooves characterization is supplied in the supplementary information Fig. S2.

As may be verified in Fig. 5c, not only each groove maintained a uniform width (within 10%) and a constant depth (within 15%) along their entire lengths, but stability was also achieved across all the grooves, with the five structures presenting similar widths and depths despite the tooth surface position variation along the processing path. This demonstrates that our system can machine reproducible structures and compensates deviations in the surface profile.

### Crater ablation evolution monitoring

**Figure 6:**
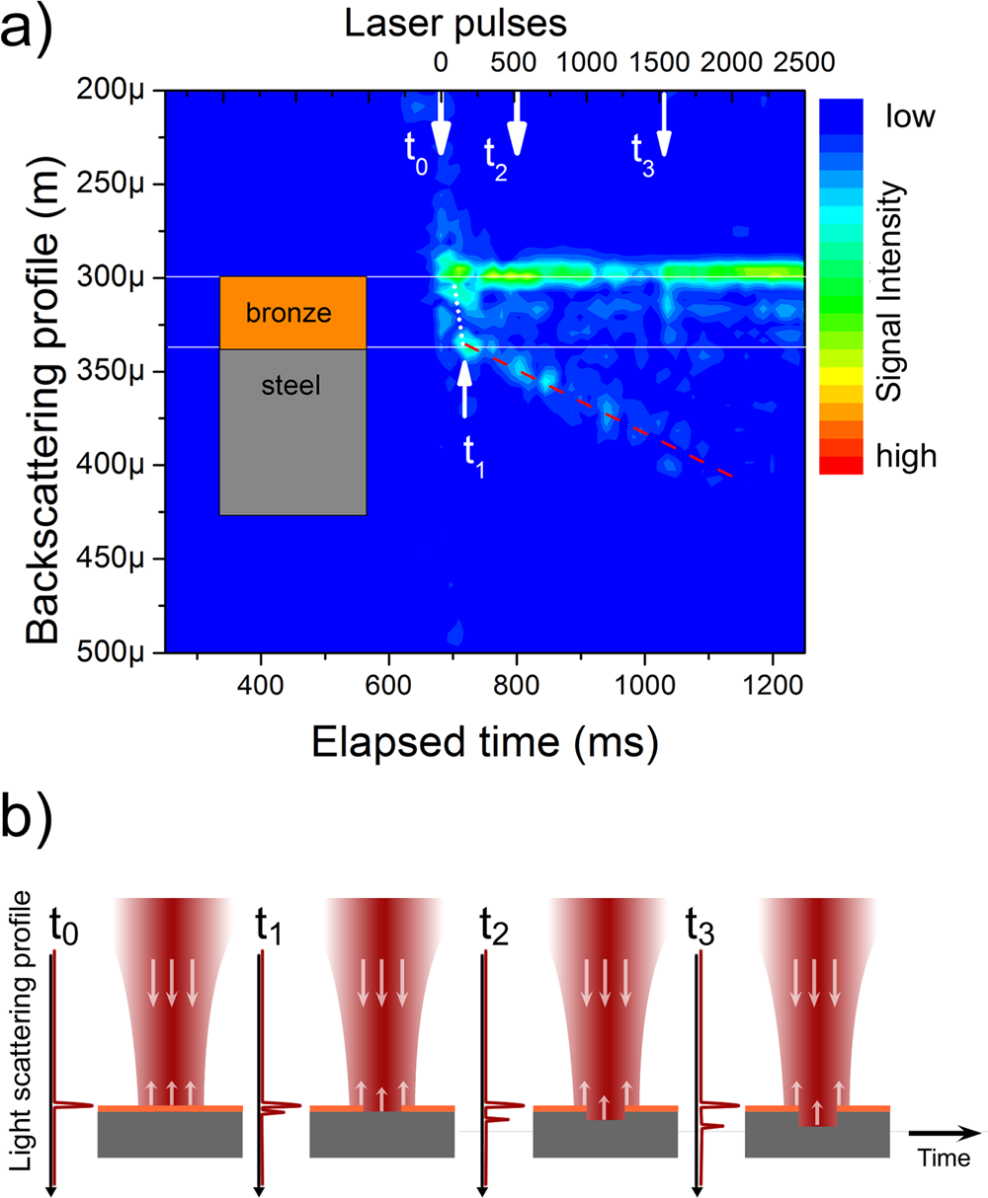
(**a**) Optical scattering profile as function of time or FLP (4 kHz rep. rate) for a dual layered metal sample. As machining starts (t_0_) the system identifies the sample surface and bronze is rapidly etched. The laser, then, reaches the steel layer (t_1_). The system keeps track of the crater evolution (t_2_ and t_3_). (**b**) An illustration of the process.

Using the same system, the results obtained for crater evolution monitoring are shown in Fig. 6a, plotting the measured surface position as a function of time for the metal sample. The surface is initially identified at 300 µm (at t_0_, around 700 ms, when machining started). One can see that the system was able to show the surface being etched away by the ablation process due to the changes in its LCI profile, akin to an Optical Coherence Tomography^8,13,14^ measurement. The monitoring was performed in real time.

After the removal of the first 35 μm from the surface, the ablation rate decreased, as can be noted by the slope change in the dashed lines, shown for reference. We hypothesize that this behavior is due to two different phenomena. One of them is the different ablation rates of bronze (plating of the coin) and steel, which is corroborated by the fact that the bronze layer thickness is 34 µm (measured by optical microscopy). Once the bronze layer is removed from the surface (t_0_–t_1_ in Fig. 6a), the machining removal rate decreases for the steel (t_1_–t_3_). This is further exacerbated by the second phenomenon: less light reaches the center of the crater as it grows deeper, since part of the beam (with intensity below the ablation threshold) gets scattered by its walls and backscatterd by the edges^15^, which is why the first surface is still visible on the LCI signal throughout the experiment (Fig. 6b).

To better understand the behavior change between t_0_–t_1_ and t_1_ onwards, the removing rates for steel and copper (major constituent of bronze at 95%) were evaluated in a complementary study. Samples of both materials were weighted using an analytical scale and a 1 cm^2^ area was laser machined (same laser system: 75 µJ pulse energy; 75 mm EFL; 20 min duration); the samples were weighted once more and the mass difference (before and after ablation) was determined. Steel presented 25% less material removed than copper, further suggesting a smaller ablation rate on steel, as observed after t_1_.

## Conclusion

We demonstrated a LCI setup that uses the same light source to machine and monitor the sample’s surface. The system kept the surface inside the focal range of the laser for most of the experiment, which resulted in a stable width of the etched grooves, even for surface height variations in excess of 1000 μm, many times greater than the beam confocal parameter. One of the advantages of the presented method is that the measurement is performed at the very same spot where the ablation takes place and it does not require an additional light source to perform the interferometry measurements. Another outcome from the setup was the demonstration that it is possible to monitor the crater evolution and, as consequence, derive the ablation rate in real time. There are demonstrations on the literature of similar results, based on different phenomena, however they have limitations for real world applications, such as requiring the perforation samples^11^, or being dependent on normal angle of incidence^16,17^. The proposed setup, by using LCI measurements, enables a wider range of applications. Nevertheless, to achieve better results and faster machining velocity, three improvements should be investigated: (1) engineered optics to enhance collection of light from the sample, retrieving a stronger signal; (2) triggered spectrometer, to synchronize the collection with the laser pulses and (3) faster processing and software refinements.

As perspectives, the system could use sub ablative intensities in order to map surfaces or even make tomographic images of biological tissues before starting the ablation, since the setup is identical to OCT or white light profilometers. For transparent samples, the system could be calibrated to have the focal spot below the surface, allowing, e.g., to create waveguides in curved glasses^18^ or to perform cornea surgery.

## Supplementary information

Supplementary Information
