# Supplementary material for: Focus Tracking System for Femtosecond Laser Machining using Low Coherence Interferometry
Source: Sci Rep. 2019 Mar 12;9:4167. doi: 10.1038/s41598-019-40749-6 (PMC6414527; doi:10.1038/s41598-019-40749-6)
Supplement: Supplementary file 1 — Supplementary Information [file 41598_2019_40749_MOESM1_ESM.pdf]

Supplementary Information to: Focus Tracking System for Femtosecond Laser Machining using Low Coherence Interferometry

Marcus Paulo Raele*, Lucas Ramos De Pretto, Wagner de Rossi, Nilson Dias Vieira Jr.and Ricardo Elgul Samad.

1. Machined surface representation

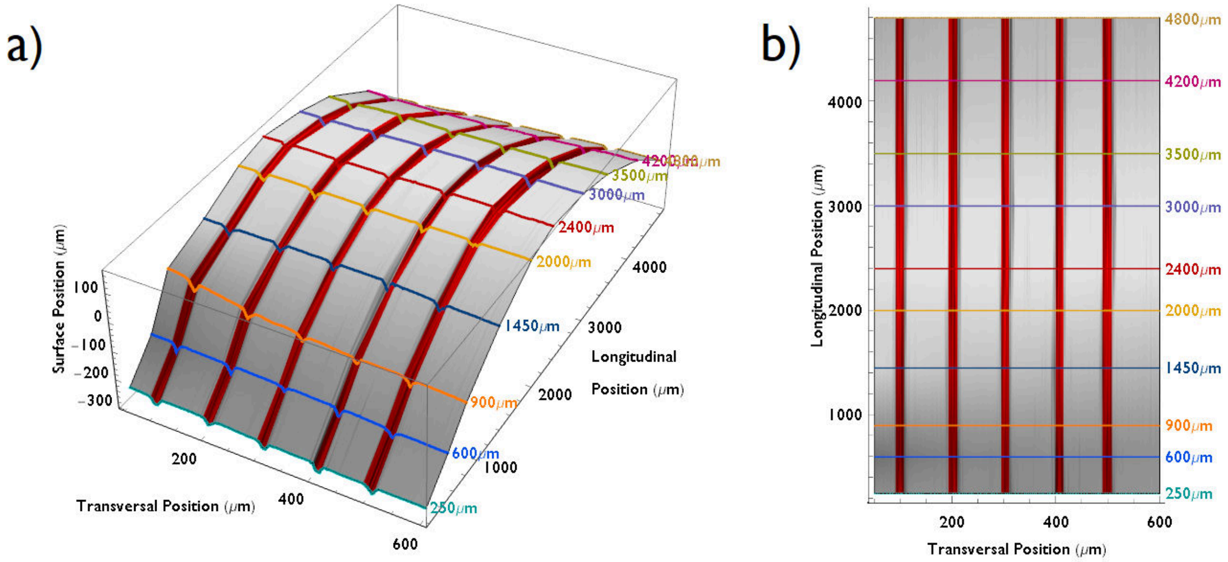

Figure S.1: Representation of the tooth surface machined with the active focus tracking system. a) 3D view of the surface depicting the grooves (highlighted in red) and the transversal profiles shown in Figure 5a of the article; the profiles colors follow the ones shown in Figure 5. b) upper view of the surface shown in (a).

2. Grooves characterization

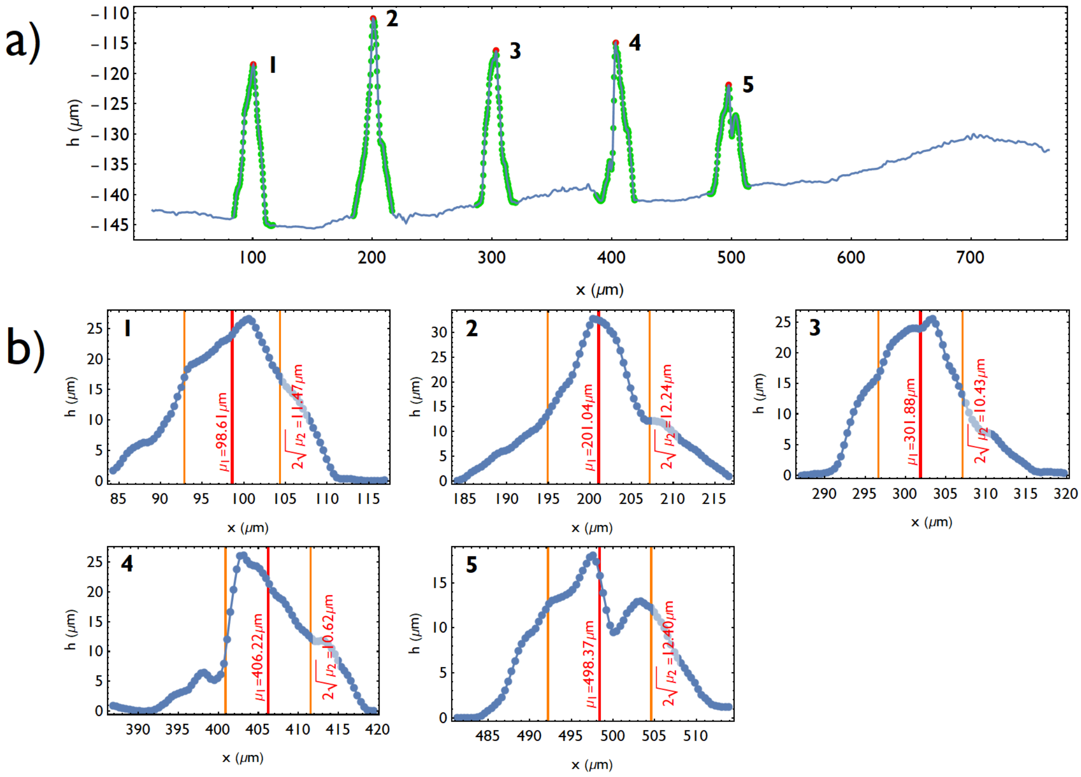

Figure S.2: a) inverted transversal profile of the surface used to find each groove and determine its width and depth. b) Selected points for each groove, with their minimal value subtracted, showing their first moment, μ1, (red line) and full width given by $2\sqrt{\mu_2}$ (limited by orange lines).

To determine the grooves' width and depth, a routine was written in Mathematica® to read a profile (one of the transversal profiles shown in Figure 5a of the article) and perform these steps:

1. Invert the profile (blue line, Figure S.2a) and find its peaks (red points, Figure S.2a), associating each one with a groove. The inversion is necessary because the software has a routine to find peaks instead of valleys (grooves);
2. Select a number of points around each peak, reaching its base (green points, Figure S.2a), and subtract the minimum height value from all points;
3. Calculate the depth, center and width for each groove, from the selected points for each peak. The groove depth is the peak height; its center is given by the point's first moment μ_1 and its full width is $2\sqrt{\mu_2}$, with μ_2 being its second moment. These moments were calculated according to equations (3) and (4) of the article using summations replacing the integrals. Each graph in Figure S.**2**b indicates the groove first moment by a red line and limits its full width by 2 orange lines with their values displayed.

3. Ablation rates for steel and copper

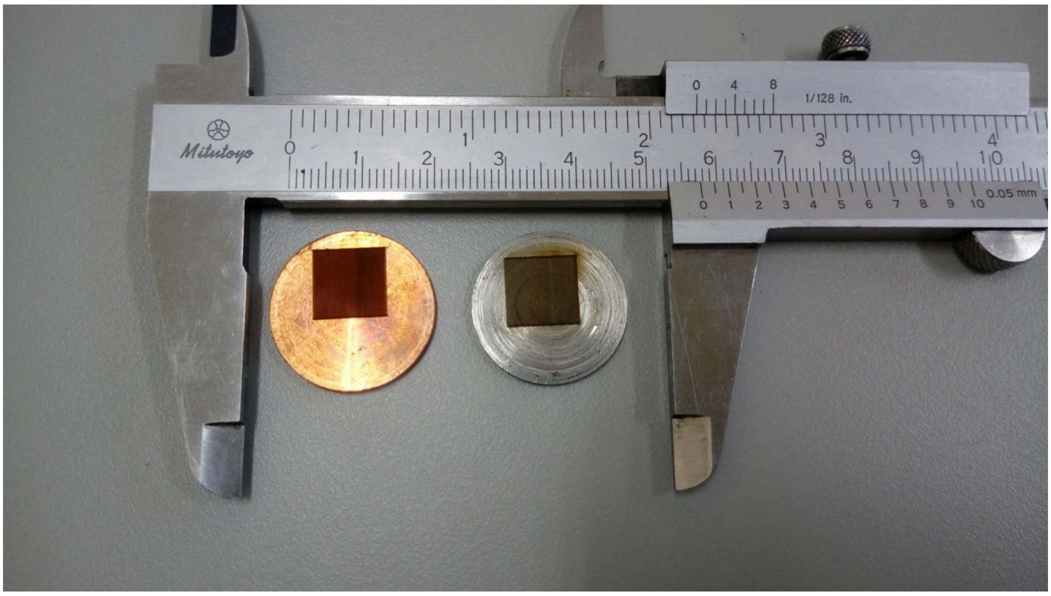

Figure S.3: Samples of copper and steel (left and right, respectively), were weighted by an analytical scale before and after ablation following identical protocols: 1cm^2 ablated area, 20 min (4 kHz repetition rate, 75 µJ pulse energy and 75 mm EFL lens). For copper the initial weight of 6.3695g changed to 6.3651g (4.4 mg ablated) and for steel it went from 4.3108g to 4.3073g (3.5 mg). This results in 25% more mass removed in the copper sample.
